# Phylogeny and mycotoxin profiles of pathogenic *Alternaria* and *Curvularia* species isolated from date palm in southern Tunisia

**DOI:** 10.3389/fmicb.2022.1034658

**Published:** 2022-11-07

**Authors:** Amal Rabaaoui, Mario Masiello, Stefania Somma, Francesco Crudo, Chiara Dall’Asta, Laura Righetti, Antonia Susca, Antonio Francesco Logrieco, Ahmed Namsi, Radhouane Gdoura, Stefaan P. O. Werbrouck, Antonio Moretti

**Affiliations:** ^1^Department of Plants and Crops, Faculty of Bioscience Engineering, Ghent University, Ghent, Belgium; ^2^Institute of Sciences of Food Production, National Research Council, Bari, Italy; ^3^Department of Food and Drug, University of Parma, Parma, Italy; ^4^Laboratoire de Phytopathologie, Centre Régional de Recherches en Agriculture Oasienne, Degache, Tunisia; ^5^Laboratory of Toxicology-Microbiology and Environmental Health, Department of Biology, University of Sfax, Sfax, Tunisia

**Keywords:** *Curvularia spicifera*, *Alternaria* section, *Ulocladioides* section, fumonisins, *Deglet Nour*

## Abstract

Date palm (*Phoenix dactylifera* L.), is a widely cultivated crop across North Africa, with about 300 thousand tons of fruits produced per year, in Tunisia. A wide range of fungal pathogens has been associated with leaf spots of date palm, *Alternaria* species being the most frequently reported. Symptomatic leaves of *Deglet Nour* variety were randomly collected in six localities in Tunisia. We used a polyphasic approach to identify 45 *Alternaria* and five *Curvularia* strains isolated from date palm, confirming their pathogenicity. Sequencing of *allergen Alt-a1*, *glyceraldehyde-3-phosphate dehydrogenase* (*gpd*) and *calmodulin* genes allowed us to group 35 strains in *Alternaria* Section, and 10 strains in *Ulocladioides* section. Based on sequencing analyses of *Internal Transcribed Spacer*, *gpd* and *elongation factor* genomic regions, all *Curvularia* strains were identified as *Curvularia spicifera*. All *Alternaria* and *Curvularia* species tested on date palm plantlets proved to be pathogenic, fulfilling Koch’s postulates. Although no significant differences were observed among the species, the highest mean disease severity index was observed in *A. arborescens*, while the lowest corresponded to *C. spicifera*. The capability of these strains to produce mycotoxins *in vitro* was evaluated. None of the *A. consortialis* strains produced any known *Alternaria* mycotoxin, whereas more than 80% of the strains included in *Alternaria* section *Alternaria* produced variable amounts of multiple mycotoxins such as alternariol, alternariol monomethyl ether, altenuene, tenuazonic acid and tentoxin. *Curvularia spicifera* strains produced detectable traces of fumonisins B. This work reports a first comprehensive multidisciplinary study of mycotoxigenic *Alternaria* species and *C. spicifera* associated with leaf spot disease on date palm.

## Introduction

Date palm (*Phoenix dactylifera* L.) is a crop widely cultivated across North Africa, from Morocco to Egypt. With about 300 thousand tons of fruits produced per year, half of which is exported (FAOSTAT, 2020), date palm occupies a strategic place in the socio-economic stability of the oasis agro-system in semiarid and arid regions in Tunisia, with about 10% of the population depending on this crop (Ben-Amor et al., 2015). Although Tunisian traditional oases play a crucial role in the maintenance of several ancient date palm cultivars, human selection, for increasing productivity and fruit size and to improve organoleptic characteristics, has led to monoculture plantation in modern oases. Indeed, nowadays, *Deglet Nour* is the most popular and most commonly cultivated variety in Tunisia (Hamza et al., 2015). Several abiotic and biotic factors, such as fungal pathogens, can compromise monoculture date palm cultivation (Hamza et al., 2015).

Wilt, leaf spots, apical dry leaf, black scorch, root rots and fruit rots are diseases commonly found in date palms. Fungal pathogens belonging to the genera *Graphiola*, *Pestalotia*, *Microsphaerella*, *Nigrospora* and *Phoma* have been isolated from date palms with symptoms of leaf spot or apical dry leaf (Abass et al., 2007; El-Deeb et al., 2007; Al-Sheikh, 2009; Abass, 2013; Khairi, 2015). In addition, *Alternaria* species are reported as the most important common foliar pathogens (Al-Nadabi et al., 2018, 2020) and several studies worldwide also reported the occurrence of a wide range of *Curvularia* species on palms showing leaf spot symptoms (Abass et al., 2007; Al-Nadabi et al., 2020; Arafat et al., 2021).

In particular, *Alternaria alternata* is the most important *Alternaria* species detected in all date palm cultivated areas. However, other *Alternaria* species also have been reported from symptomatic date palm leaves, such as *A. burnsii* and *A. arborescens*, in Oman (Al-Nadabi et al., 2018); *A. chlamydospora* and *A. radicina* in Iraq (Abass et al., 2007; Khudhair et al., 2015); *A. bokurai*, *A. arborescens* and *A. mali* in Tunisia (Ben Chobba et al., 2013; Namsi et al., 2021). Most of these reports were based only on morphological identification. The uncertain species boundaries based on morpho-taxonomy, the environmental factors influencing the morphological traits, the high similarity between some species and the presence of several strains with intermediary traits, are all aspects that could cause many errors in *Alternaria* identification. Therefore, a different approach is needed (Andrew et al., 2009; Somma et al., 2019).

Based primarily on morphological characters, Simmons organized the genus complexity of *Alternaria* by launching a species-group concept and identified more than 270 *Alternaria* morpho-species.

Important taxonomic revision within the genus *Alternaria* have been carried out through molecular studies, by using a multi-locus gene sequence approach. *Alternaria* morpho-species were phylogenetically analyzed and defined first as species-groups, then elevated to the status of sections (Lawrence et al., 2013, 2014; Woudenberg et al., 2015). According to this taxonomic revision, several morpho-species have been synonymized, and *A. alternata* has been divided into more than 35 species, including species previously belonging to closely related genera (Woudenberg et al., 2015; Somma et al., 2019).

For a correct identification of *Alternaria* species, a polyphasic approach, based on morphological characterization, genetic analyses and production of secondary metabolites, has been proposed. Indeed, some *Alternaria* species are known for the production of a wide range of secondary metabolites, including mycotoxins and host/non host specific toxins (Akimitsu et al., 2014; Meena and Samal, 2019).

The most important *Alternaria* mycotoxins are alternariol (AOH), alternariol monomethyl ether (AME), altenuene (ALT), and tenuazonic acid (TA; Logrieco et al., 2009; Da Cruz Cabrala et al., 2016). Based on several *in vitro* and *in vivo* assays, toxicity, mutagenicity and genotoxicity of these metabolites have been demonstrated (Lehmann et al., 2006; Ostry, 2008; Zhou and Qiang, 2008), and the risks for human and animal health have been studied (Ostry, 2008; Alexander et al., 2011).

In addition, several phytotoxins, among which some are considered host specific toxins, are known to be produced by species in the genus *Alternaria* (Alexander et al., 2011; Escrivá et al., 2017). Some of them can play a crucial role in pathogenicity processes, acting as virulence and colonization factors, such as AAL toxin, which plays a key role in pathogenesis of stem canker in tomato, caused by *A. arborescens* (Prasad and Upadhyay, 2010).

Indeed, it must be underlined that AAL toxin is chemically considered as part of the fumonisin family, a group of mycotoxins commonly produced by several *Fusarium* species (Desjardins, 2006). Fumonisins are classified by the International Agency of Research on Cancer, as group 2B, possible carcinogen to humans, since they have been related to esophageal cancer (International Agency for Research on Cancer - IARC, 2002). In addition, fumonisins have been associated with several animal diseases, such as porcine pulmonary edema, and equine leucoencephalomalacia (Desjardins, 2006).

The aims of this study were: (i) to identify a set of *Alternaria* and *Curvularia* strains isolated from leaves showing symptoms of leaf spot disease, by using a polyphasic approach; (ii) to assess their pathogenicity on date palm plantlets of the most common Tunisian variety *Deglet Nour*; (iii) to define the mycotoxin profiles of representative strains belonging to *Alternaria* and *Ulocladioides* sections; (iv) to assess the capability of *C. spicifera* strains to produce mycotoxins.

## Materials and methods

### Origin of the samples and fungal isolation

During the years 2017–2019, an in-depth survey was carried out in the modern and ancient oases of the Djérid, in south-western Tunisia: IBN Chabbat, Mides, Dgeuch, Tozeur, Nafta, El-Hamma, Hezoua. For each area, 20 samples of date palm leaflets showing characteristic symptoms of Leaf Spot Disease were randomly collected from six different plantations.

After a surface-disinfection with 2% sodium hypochlorite solution for 2 min, 70% ethanol for 30 s, and washing twice with distilled sterilized water for 1 min, portions of leaflet tissues were dried on sterile filter paper in a laminar flow cabinet. Small pieces of about 2×2 mm, taken from the margin of the symptomatic tissues, were placed on Potato Dextrose Agar (PDA) amended with 100 mg L^−1^ of streptomycin sulphate salt and 50 mg L^−1^ of neomycin. Petri dishes were incubated at 25 ± 1°C for 7 days under an alternating light/darkness cycle of 12 h photoperiod. After incubation, fungal colonies originated from plant tissues were transferred to new PDA plates and then purified by using the single spore isolation technique.

A set of 45 representative *Alternaria* strains and 5 *Curvularia* strains (Table 1), were selected and stored at-80°C in 10% glycerol, as suspensions of conidia and mycelium, for further molecular and chemical analyses.

**Table 1:** *Alternaria* and *Curvularia* strains isolated from date palm, in Tunisia, during 2017–2019.

Strain	Fungal species	Geographical origin	Year of isolation	Accession number		
				*Alt-a1*	*CaM*	*gpd*
A3	*Alternaria consortialis*	IBN Chabbat	2017	ON688353	ON688398	ON688443
A6		IBN Chabbat	2017	ON688346	ON688391	ON688436
A36		IBN Chabbat	2018	ON688347	ON688392	ON688437
A38		IBN Chabbat	2018	ON688345	ON688390	ON688435
Alt1553		Mides	2018	ON688348	ON688393	ON688438
Alt1559		Mides	2019	ON688350	ON688395	ON688440
Alt1565		Mides	2019	ON688349	ON688394	ON688439
Alt1568		Dgeuch	2017	ON688352	ON688397	ON688442
A33		Dgeuch	2017	ON688354	ON688399	ON688444
Alt1571		Tozeur	2018	ON688361	ON688396	ON688441
Alt1569	*Alternaria arborescens* ^A1^	IBN Chabbat	2017	ON688315	ON688360	ON688405
		IBN Chabbat	2017	ON688310	ON688355	ON688390
A16						
A8		Mides	2017	ON688311	ON688356	ON688401
A37		Mides	2017	ON688321	ON688366	ON688411
Alt1555		Dgeuch	2017	ON688318	ON688363	ON688408
Alt1550		Dgeuch	2018	ON688316	ON688361	ON688406
Alt1552						
		Dgeuch	2018	ON688319	ON688364	ON688409
Alt1558		Tozeur	2017	ON688313	ON688358	ON688403
Alt1561		Tozeur	2017	ON688320	ON688365	ON688410
A14		Nafta	2017	ON688312	ON688357	ON688402
Alt1575		El-Hamma	2018	ON688314	ON688359	ON688404
Alt1570		Hezoua	2018	ON688317	ON688362	ON688407
Alt1551	*Alternaria tenuissima* ^A2^	IBN Chabbat	2017	ON688338	ON688383	ON688428
A4alt		IBN Chabbat	2018	ON688328	ON688373	ON688418
Alt1557		IBN Chabbat	2018	ON688330	ON688375	ON688420
Alt1576		IBN Chabbat	2018	ON688331	ON688376	ON688421
Alt1580		IBN Chabbat	2019	ON688326	ON688371	ON688416
Alt1567		Mides	2017	ON688333	ON688378	ON688423
Alt1577		Mides	2017	ON688322	ON688367	ON688412
A23 Alt1560		Mides	2019	ON688327	ON688372	ON688417
Alt1579		Mides	2019	ON688323	ON688368	ON688413
		Mides	2019	ON688324	ON688369	ON688414
A26		Tozeur	2017	ON688329	ON688374	ON688419
A30		Tozeur	2017	ON688334	ON688379	ON688424
Alt1554		Hezoua	2017	ON688332	ON688377	ON688422
A13		Nafta	2017	ON688325	ON688370	ON688415
Alt1556		Nafta	2017	ON688335	ON688380	ON688425
Alt1564		Dgeuch	2017	ON688337	ON688382	ON688427
Alt1574		El-Hamma	2017	ON688336	ON688381	ON688426
Alt1562	*Alternaria alternata* ^A3^	Mides	2017	ON688343	ON688388	ON688433
A12		Mides	2018	ON688340	ON688385	ON688430
A19		IBN Chabbat	2018	ON688341	ON688386	ON688431
		IBN Chabbat	2018	ON688344	ON688389	ON688434
Alt1563						
Alt1566		Dgeuch	2017	ON688342	ON688387	ON688423
Alt1578		Dgeuch	2018	ON688336	ON688384	ON688429
ITEM18913	*Curvularia spicifera*	Nafta	2017	ON673959	ON688453	ON688448
ITEM18910		IBN Chabbat	2018	ON688957	ON688451	ON688446
ITEM18911		IBN Chabbat	2018	ON688960	ON688454	ON688449
ITEM18912		Tozeur	2018	ON688956	ON688450	ON688447
ITEM18909		Tozeur	2019	ON688958	ON688452	ON688445

A1, *Alternaria* section sub-clade A1; A2, *Alternaria* section sub-clade A2; A3, *Alternaria* section sub-clade A3.

### DNA extraction and molecular analyses

Forty-five *Alternaria* and 5 *Curvularia* monoconidial strains were first cultured on PDA medium, and after 2–3 days of incubation, 5 mycelial plugs from the margins of actively growing colonies were transferred on cellophane disks overlaid on PDA plates. After 3 days of incubation at 25°C, mycelium of each strain was collected by scraping and was lyophilized. For each strain, DNA was extracted and purified from powdered lyophilized mycelium (10–15 mg) by using the “Wizard Magnetic DNA Purification System for Food” kit (Promega Corporation, Madison, WI, United States), according to the manufacturer’s protocol. Integrity of DNA was checked by electrophoretic analysis on 0.8% agarose gel and by comparison with a standard DNA (1 kb DNA Ladder, Fermentas GmbH, St. Leon-Rot, Germany); quantity was evaluated by Thermo-Scientific Nanodrop (LabX, Midland, ON, Canada).

The informative genes *Allergen Alt-a1* (*Alt-a1*), *glyceraldehyde 3-phosphate dehydrogenase* (*gpd*), and *calmodulin* (*CaM*) were selected for the molecular characterization and for building a reliable phylogenetic relationship among *Alternaria* strains, using a multi-locus sequence approach.

For each gene, PCR mixture (15 μl) containing approximately 15–20 ng of DNA template, 1.5 μl (10X) PCR solution buffer, 0.45 μl of each primer (10 mM), 1.2 μl dNTPs (2.5 mM), and 0.125 μl of Hot Start Taq DNA Polymerase (1 U/μL; Fisher Molecular Biology, Trevose, Pennsylvania, US) was amplified. Amplification of *Alt-a1* and *gpd* genes was performed with the primer pairs alt-for/alt-rev (Hong et al., 2005), gpd1/gpd2 (Berbee et al., 1999) using PCR reaction parameters as reported by Masiello et al. (2019), *CaM* gene was amplified using the PCR conditions reported by Habib et al. (2021).

Elongation factor (tef), Internal Trascribed Spacer (*ITS*), *gpd* and *CaM* genes were amplified for the molecular characterization of the five *Curvularia* strains. In particular, *gpd* and *CaM* genes were amplified using the same primers and PCR conditions used for *Alternaria* strains. Internal transcribed spacer was amplified with ITS4/ITS5 primers pair (White et al., 1990) setting up the termocycler at 95°C for 3 min, 40 cycles at 95°C per 30 s, 52°C for 30s and 72°C for 50 s, a final extension at 72°C for 7 min. Elongation factor-*1a* gene was amplified using PCR conditions reported by Somma et al. (2019).

For each reaction, a no-template control was included to ascertain the absence of contamination. The PCR products, stained with GelRed^®^ (GelRed^®^ Nucleic Acid Gel Stain, 10000X, Biotium Inc., Fremont, California, United States) were visualized with UV after electrophoretic separation in 1X TAE buffer, on 1.5% agarose gel and sized by comparison with 100 bp DNA Ladder (Invitrogen, Thermo Fisher Scientific, Carlsbad, California, United States).

### Sequencing and phylogenetic analysis

Each PCR product was purified with the enzymatic mixture Exo/FastAP (Exonuclease I, FastAP thermosensitive alkaline phosphatase, Thermo Scientific, Lithuania, Europe) and then sequenced with Big Dye Terminator Cycle Sequencing Ready Reaction Kit (Applied Biosystems, Foster City, CA, United States), according to the manufacture’s recommendations for both strands of each gene. The fragments were purified by filtration through Sephadex G-50 (5%; Sigma-Aldrich, Saint Louis, MO, United States) and sequenced in “ABI PRISM 3730 Genetic Analyzer” (Applied Biosystems, Foster City, CA, United States). Partial sequences were assembled using the BioNumerics v. 5.1 software (Applied Maths, Inc., Austin, Texas, United States). Phylogenetic trees of concatenated gene sequences were generated by using maximum likelihood statistical method and bootstrap analysis (1,000 replicates, removing gaps) with MEGA7 (Kumar et al., 2016).

The bootstrap analysis (Felsenstein, 1985) was conducted to determine the confidence of internal nodes using a heuristic search with 1000 replicates, removing gaps. Gene sequences of the reference strains *A. alternata* strain EV-MIL-31, *A. alternata* strain ATCC 34957, *A. alternata* strain MOD1-FUNGI5, *A. arborescens* strain FERA 675, *A. mali* strain EGS38.029, *A. tenuissima* strain FERA 1166, *A. atra* strain MOD1-FUNGI7, *A. consortialis* strain JCM 1940 were downloaded from the National Center for Biotechnology Information (NCBI) and included in the phylogenetic analysis.

Sequences of *Curvularia* strains were analysed together with reference sequences of *C. spicifera* CBS 274.52, *C. hawaiiensis* BRIP 10971, *C. australiensis* CBS 172.57, *C. lunata* CBS 730-96, *C. perotidis* CBS 350.90 as reported by Manamgoda et al. (2012).

### Mycotoxin analyses

Thirty-two *Alternaria* strains and the five *Curvularia* strains were analysed for their capability to produce mycotoxins *in vitro*. Was inoculated, Three small plugs from 1-week-old colonies of each fungal strain were used to inoculate 30 g autoclaved rice with 40% moisture in 250 ml of flasks. Flasks were incubated for 21 days at 25°C in darkness and then the samples were finely ground with an Oster Classic grinder (220–240 V, 50/60 Hz, 600 W; Madrid, Spain).

For mycotoxin analyses, samples were prepared according to Malachová et al. (2014) procedure. Briefly, 0.5 g of ground cereal was stirred for 90 min at 200 strokes/min on a shaker with 2 ml of acetonitrile/water (80/20, v/v) mixture acidified with 0.2% of formic acid and then centrifuged for 10 min at 14,000 rpm. 1 μl of supernatant was injected into LC–MS.

Mycotoxin standards of AOH, AME, ALT, ATX-I, TA, TEN, and fumonisins were obtained from Romer Labs (Tulln, Austria).

LC–MS grade methanol, and acetonitrile were purchased from Scharlab Italia srl (Milan, Italy); bidistilled water was obtained using Milli-Q System (Millipore, Bedford, MA, United States). MS-grade ammonium acetate, acetic acid and formic acid were purchased from Fisher Chemical (Thermo Fisher Scientific Inc., San Jose, CA, United States).

#### UHPLC-TWIMS-QTOF screening of mycotoxins

ACQUITY I-Class UPLC separation system coupled to a VION IMS QTOF mass spectrometer (Waters, Wilmslow, United Kingdom) equipped with an electrospray ionization (ESI) interface was employed for mycotoxin screening. Samples were injected (1 μl) and chromatographically separated using a reversed-phase C18 BEH ACQUITY column 2.1 × 100 mm, 1.7 μm particle size (Waters, Milford, MA, United States). A gradient profile was applied using water 1 mM ammonium acetate (eluent A) and methanol (eluent B) both acidified with 0.5% acetic acid as mobile phases. Initial conditions were set at 5% B, after 0.7 min of isocratic step, a linear change to 50% B in 5.8 min. 100% B was achieved in 3 min and holding for 3 min to allow for column washing before returning to initial conditions. Column recondition was achieved over 1.5 min, providing a total run time of 14 min. The column was maintained at 40°C and a flow rate of 0.4 ml/min used.

Mass spectrometry data were collected in positive electrospray mode over the mass range of *m/z* 50–1,000. Source settings were maintained using a capillary voltage, 1.0 kV; source temperature, 150°C; desolvation temperature, 600°C and desolvation gas flow, 1,000 l/h. The TOF analyzer was operated in sensitivity mode and data acquired using HDMSE, which is a data independent approach (DIA) coupled with ion mobility. The optimized ion mobility settings included: nitrogen flow rate, 90 ml/min (3.2 mbar); wave velocity 650 m/s and wave height, 40 V. Device within the Vion was calibrated using the Major Mix IMS calibration kit (Waters, Wilmslow, United Kingdom) to allow for CCS values to be determined in nitrogen. The calibration covered the CCS range from 130 to 306 Å2. The TOF was also calibrated prior to data acquisition and covered the mass range from 151 Da to 1,013 Da. TOF and CCS calibrations were performed for both positive and negative ion mode. Data acquisition was conducted using UNIFI 1.8 (Waters, Wilmslow, United Kingdom).

Mycotoxin identification was performed by comparison of retention time, fragmentation pattern and collision cross sections with the standard collect in our UNIFI library, created by running a mix of standards with the same analytical method. Quantification of target analytes was performed using calibration standards in the range 0.1–2 mg kg^−1^.

### Pathogenicity assay

Pathogenicity assays were carried out by using 39 fungal strains, selected among all *Alternaria* phylogenetic clades, and 3 out of the 5 *Curvularia* strains (Table 1). The strains were grown for 7–10 days at 25°C on PDA, under a 12 h light/darkness photoperiod, to favor fungal sporulation. For each strain, the inoculum was prepared by flooding the agar plate surface with 10 ml of sterile distilled water (SDW) and scraping with a spatula. Conidial suspensions were filtered through four layers of cheese cloth and adjusted at a final concentration of 10^6^ conidia mL^−1^.

The pathogenicity tests were performed on healthy date palm plantlets (cv. *Deglet Nour*), regenerated from direct somatic embryogenesis, derived from shoot-tip explants. Before pathogenicity test, the plantlets were acclimatized in greenhouse under suitable controlled conditions, for 6-months.

The plantlets were of 25–30 cm length, having 4–5 leaves, and grown in large pots (12 cm diameter and 18 cm height filled with planting medium peat/perlite 2:1 (v/v)). Crown area of each plantlet was sprayed with 4 ml of conidial suspension by using hand operated compressed air sprayer. Pathogenicity of each fungal strain was assessed in triplicates and plantlets sprayed only with SDW were used as control. To favor fungal development, all plantlets were watered and enclosed in plastic bags for 3 days in a greenhouse set at 25 ± 2°C, 80% RH, 12 h light/darkness photoperiod. After inoculation, the first visual assessment was carried out after 10 days and then weekly for 3 months.

The severity of disease symptoms was calculated according to Bhat et al. (2013), with 6 disease severity classes from 0 (healthy plantlets) to 5 (disease symptoms on the 75–100% of the plantlet area). Intermediate disease symptoms were assigned to 4 classes (1 = 0–1 spot and yellowing of about 0.1–10% of the plantlet area; 2 = 1–3 spots, with yellowing of 11.1–25% of the plantlet area; 3 = 4–6 spots, with yellowing of 26–50% of the plantlet area; 4 = 7–9 spots, with yellowing up to 75% of the plantlet area). The Disease Severity Index was calculated for each plantlet by McKinney’s formula


DSI=(∑vn)/(NV)×100

Where *n* is number of palm plantlets per class, *v* is the numerical value of each class, *N* is the total number of plantlets and *V* is the highest class value.

Differences in the disease severity incidence between isolates were tested for statistical significance with Kruskal–Wallis tests using Statistical Package for the Social Sciences (SPSS) v. 27.0 software, with significance level (*P*) of 0.05. To confirm Koch’s postulates, pieces of leaves, where symptoms of the disease appeared, were sterilized on the surface and the fungi were re-isolated.

## Results

### Phylogenetic analyses

**Figure 1:**
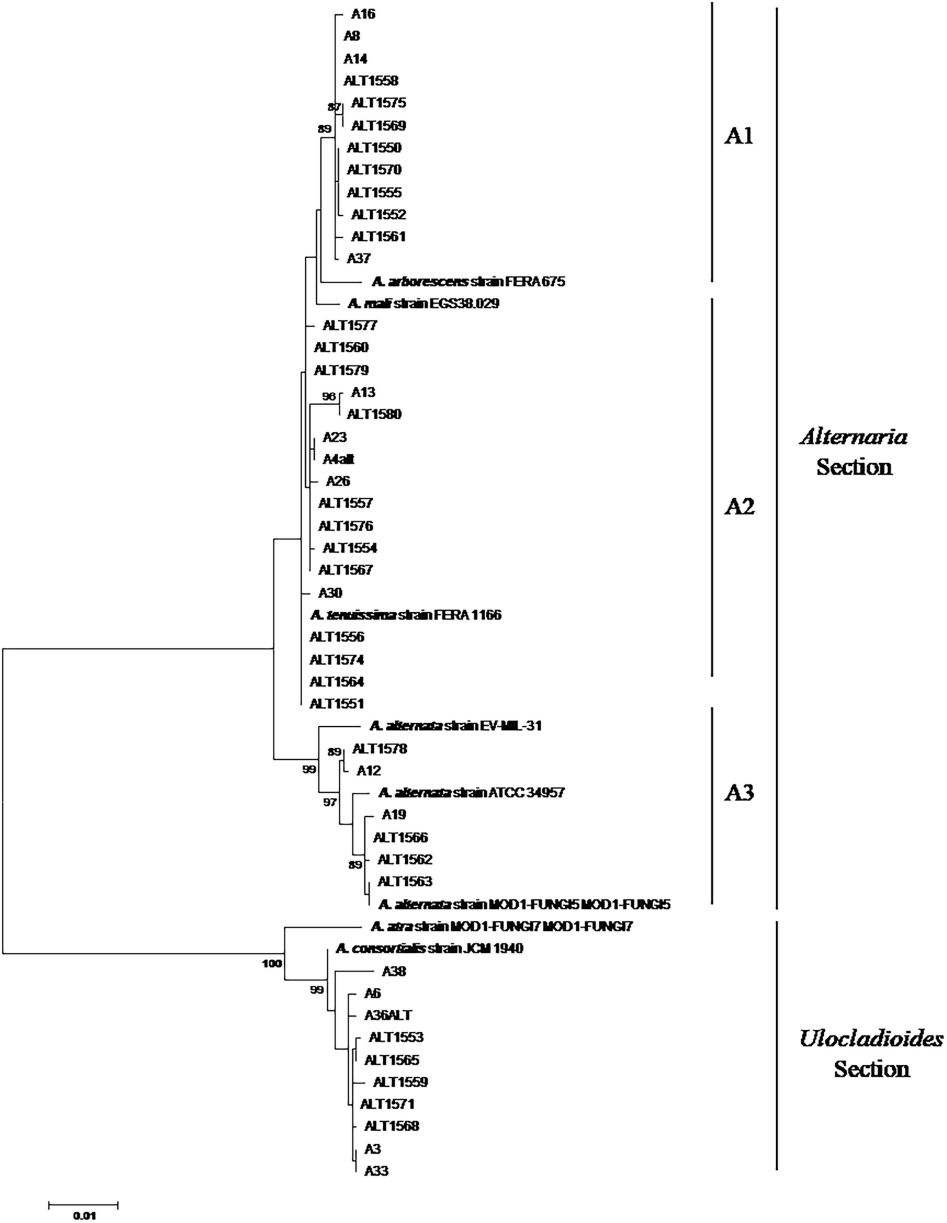
Phylogenetic tree generated by using the Maximum Likelihood method of combined *CaM*, alt-a1, and *gpd* gene sequences of 45 *Alternaria* strains isolated from date palm, in Tunisia. Numbers on branches are bootstrap values (>70) based on 1,000 replicates.

Phylogenetic relationships among 45 *Alternaria* strains were studied at the genetic level by amplifying fragments of three different genes: *CaM*, *Alt-a1*, and *gpd*. To further resolve the identity of the *Alternaria* strains, the sequences of each gene were aligned and cut at the ends to analyse a common fragment for all the strains. In particular, 675, 485, and 590 nucleotide sites were used for *CaM*, *Alt-a1*, and *gpd* genes, respectively. For each strain, the three gene sequences were concatenated and analysed simultaneously with species reference strains (Figure 1).

The phylogenetic analysis of the concatenated sequences of 1750 positions resulted in a phylogenetic combining dataset comprising 53 taxa, including 45 *Alternaria* field strains and 8 *Alternaria* reference sequences. The phylogenetic tree was resolved in two well-separated clades corresponding to *Alternaria* and *Ulocladioides* sections, supported by high bootstrap values (Figure 1).

Thirty-five strains (77.7%) were assigned to the *Alternaria* Section *Alternaria*. In particular, 12 strains shared a very high similarity among them and clustered with *A. arborescens* FERA 675 reference strain (Figure 1; sub-clade A1). A well-supported group clustered together the three *A. alternata* reference strains (EV-MIL-31, ATCC 34957 and MOD1-FUNGI5) and six field strains (Figure 1; sub-clade A3). The other 17 strains clustered together with *A. tenuissima* FERA1166 in a not well-supported group (sub-clade A3).

A well-supported clade (clade B), defined as *Ulocladioides* section, grouped together *A. consortialis* JCM 1940 reference strain, *A. atra* MOD1-FUNGI7 and 10 field strains (Figure 1). In particular, with the exception of A38 strain, they showed high homology among them (Figure 1).

**Figure 2:**
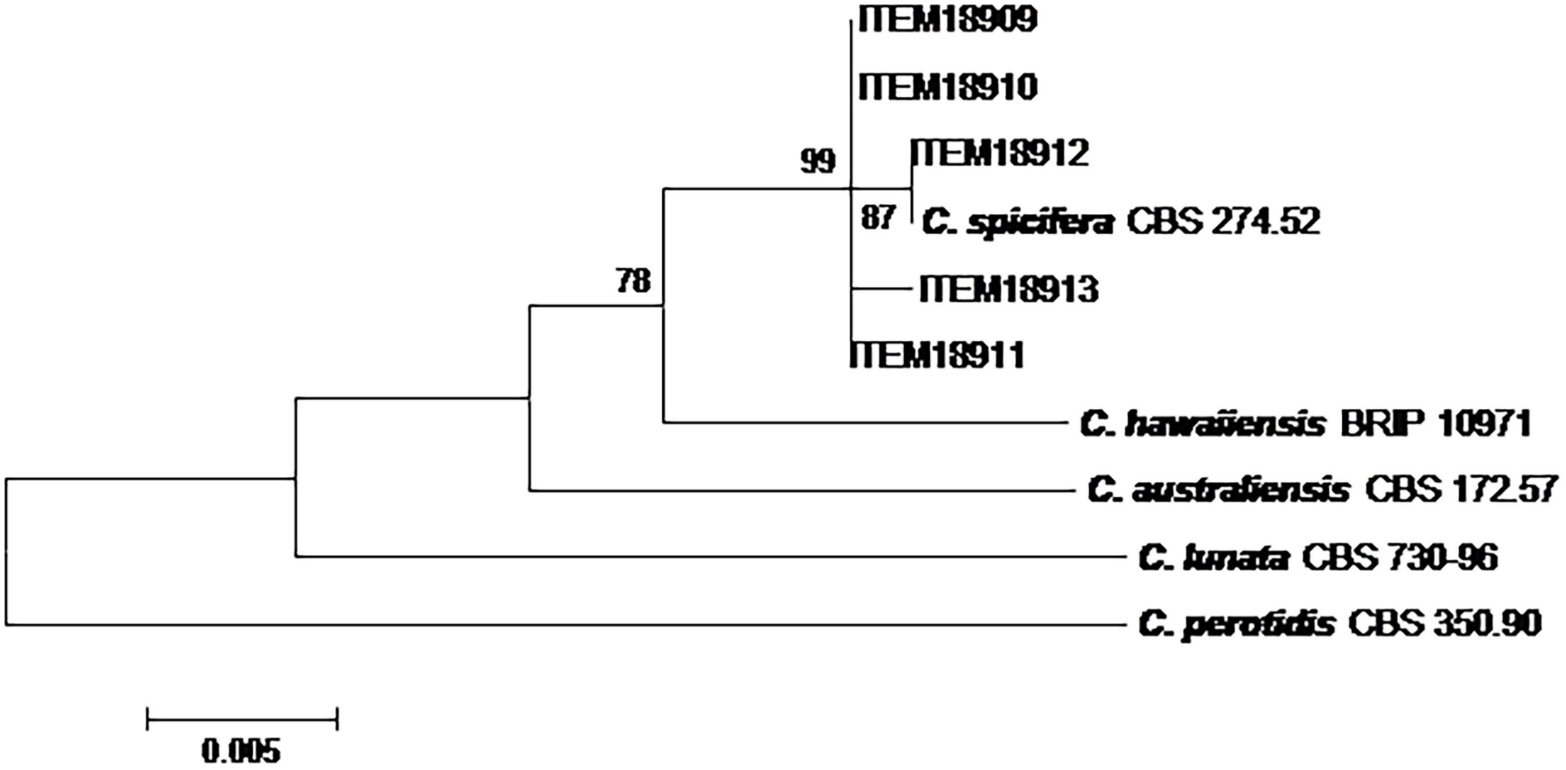
Phylogenetic tree generated by maximum likelihood analysis (bootstrap 1,000 replicates) of combined *ITS*, *gpd* and *EF-1α loci* of 5 *Curvularia* strains isolated from date palm, in Tunisia. Numbers on branches are bootstrap values (>70) based on 1,000 replicates.

The phylogenetic analysis on five *Curvularia* strains was carried out considering a concatenated sequence of 1,318 sites, including the 5 field strains, 4 *Curvularia* reference strains and *A. alternata* EGS 34.016 considered as the outgroup taxon (Figure 2). The phylogenetic tree showed that all field strains clustered together, showing high homology with *C. spicifera* CBS 274.52. They formed a well-separated group (bootstrap 99) from the other *Curvularia* species included in the analyses, genetically closely related to *C. spicifera* (Figure 2).

Sequences derived in this study were deposited in the GenBank database (Table 1).

### Mycotoxin production

Thirty-two *Alternaria* strains were selected among the phylogenetic sub-clades (Figure 1) to evaluate their capacity to produce the mycotoxins considered so far the most common ones produced by the species of *Alternaria*. The mycotoxin production of each strain and the mean values of each *Alternaria* phylogenetic sub-clade are reported in Table 2. The five *C. spicifera* strains were also analysed to investigate their capability to produce the selected mycotoxins. In detail, chemical analyses were carried out on eight *Alternaria* strains which clustered with *A. arborescens* reference strain, 11 strains that clustered with *A. tenuissima* reference strain, three strains genetically identified as *A. alternata*, 10 *A. consortialis* strains and five *C. spicifera* strains. None of the *A. consortialis* and *Curvularia* strains synthesized any of the *Alternaria* mycotoxins selected in the study. Among the strains included in the three sub-clades of *Alternaria* section, the majority of the strains were able to produce the selected mycotoxins. In particular, 41% of the strains were able to co-produce simultaneously all five mycotoxins investigated, whereas 32% of strains produced 4 mycotoxins (Table 2). In *A. arborescens* group (sub-clade A1), all strains produced AOH with values ranging between 68.6 and 974.2 μg g^−1^ (mean value 428 μg g^−1^), AME with values ranging between 31 and 372.5 μg g^−1^ (mean value 221.7 μg g^−1^), and TA with values ranging between 5.7 μg g^−1^ and 22.2 μg g^−1^ (mean value 13.5 μg g^−1^). Only 4 out of 8 strains (A8, A14, ALT1575, and A37) produced ALT with values ranging between 35.7 and 115.2 μg g^−1^ (mean value 71 μg g^−1^). TEN was produced by all strains, with the exception of the strains ALT1558 and ALT1570, with values that never exceeded 15.1 μg g^−1^ (mean value of 6.2 μg g^−1^).

**Table 2:** Mycotoxin production by *Alternaria* and *Curvularia* strains isolated from date palm in Tunisia.

Strain	Mycotoxin				
	ALT	AOH	AME	TEN	TA
*Alternaria* Section sub-clade A1 (*A. arborescens*)					
A16	n.d.	421.1	174.0	1.2	11.6
A8	35.7	519.5	321.3	1.5	20.5
A14	115.2	974.2	372.5	9.6	22.1
ALT1575	85.7	814.9	332.4	15.1	14.0
ALT1558	n.d.	68.6	31.0	n.d.	5.7
ALT1550	n.d.	203.5	132.1	6.8	11.3
ALT1570	n.d.	163.6	103.5	n.d.	5.7
A37	47.2	258.5	306.8	3.2	16.8
N. of positive/total strains	**4/8**	**8/8**	**8/8**	**6/8**	**8/8**
Frequency (%)	**50**	**100**	**100**	**75**	**100**
MIN	**35.7**	**68.6**	**31**	**1.2**	**5.7**
MAX	**115.2**	**974.2**	**372.5**	**15.1**	**22.2**
Mean value	**71**	**428**	**221.7**	**6.2**	**13.5**
*Alternaria* Section sub-clade A2 (*A. tenuissima*)					
A4ALT	14.9	16.2	30.7	13.1	3.5
A23	31.4	35.9	65.1	14.6	4.7
A26	19.2	27.8	61.6	10.7	5.5
A30	n.d.	18.8	12.3	1.0	n.d.
ALT1554	n.d.	60.4	27.2	0.3	1.4
ALT1557	n.d.	6.3	3.0	0.3	0.4
ALT1576	23.3	58.5	99.8	3.9	9.8
ALT1579	n.d.	45.8	4.7	6.2	n.d.
A13	n.d.	38.8	18.6	0.5	0.8
ALT1574	n.d.	56.3	21.5	0.5	n.d.
ALT1551	n.d.	n.d.	n.d.	0.4	n.d.
N. of positive/total strains	**4/11**	**10/11**	**10/11**	**11/11**	**7/11**
Frequency (%)	**36.4**	**91**	**91**	**100**	**63.6**
MIN	**14.9**	**6.3**	**3**	**0.3**	n.d.
MAX	**31.4**	**60.4**	**99.8**	**14.6**	9.8
Mean value	**22.2**	**36.5**	**34.4**	**4.7**	2.4
*Alternaria* Section sub-clade A3 (*A. alternata*)					
A12	n.d.	352.7	183	3.2	56.5
A19	45	35.6	91.4	0.6	6.0
ALT1563	n.d.	12	n.d.	n.d.	n.d.
N. of positive/total strains	**1/3**	**3/3**	**2/3**	**2/3**	**2/3**
Frequency (%)	**33.3**	**100**	**66.6**	**66.6**	**66.6**
MIN	**-**	**12**	**91.4**	**0.6**	n.d.
MAX	**-**	**352.7**	**183**	**3.2**	**56.5**
Mean value	**-**	**133.4**	**137.2**	**1.9**	**20.8**
*Ulocladioides* section (*A. consortialis*)					
N. of positive/total strains	**0/10**	**0/10**	**0/10**	**0/10**	**0/10**
*Curvularia spicifera*					
N. of positive/total strains	**0/5**	**0/5**	**0/5**	**0/5**	**0/5**

Data are expressed as μg/g of dried rice kernels. ALT, altenuene; AOH, alternariol; AME, alternariol monomethyl ether; TEN, tentoxin; TA, tenuazonic acid. N. of positive strains, frequency, minimum, maximum and mean values for each phylogenetic group are reported in bold.

The strains included in the *A. tenuissima* sub-clade showed a slightly lower capability to produce mycotoxins (Table 2). With the exception of Alt11551, which produced only trace amounts of TEN, all strains produced AOH with values ranging between 6.3 and 60.4 μg g^−1^ (mean value 36.5 μg g^−1^) and AME with values ranging between 3 and 99.8 μg g^−1^ (mean value 34.4 μg g^−1^). All strains produced TEN (mean values 4.7 μg g^−1^), although 8 out of 11 strains produced less than 1 μg g^−1^. Altenuene was produced by 4 out of 11 strains with values ranging between 14.9 and 31.4 μg g^−1^ (mean value of 22.2 μg g^−1^). With the exception of the strains A26, Alt1579, Alt1574 and Alt1551, TA was produced with values ranging between less than LOQ and 9.4 μg g^−1^ (mean values of 2.4 μg g^−1^).

Among *A. alternata* strains, Alt1563 produced only AOH (12 μg g^−1^), whereas the other two strains produced AOH, AME, TEN, and TA (Table 2). Only A19 strain produced ALT (45 μg g^−1^).

The mycotoxin analysis was performed using a HR-IMS instrument, taking advantage of the ion mobility CCS values library developed in house and validated over an inter laboratory and inter platform study (Righetti et al., 2018, 2020). The CCS value represents a univocal chemical feature of an analyte, offering an additional and independent point of identification with respect to the MS spectra and the retention time. The database collects the consistent CCS values for 53 mycotoxins, and it is therefore extremely useful for the retrospective qualitative analysis of samples.

**Figure 3:**
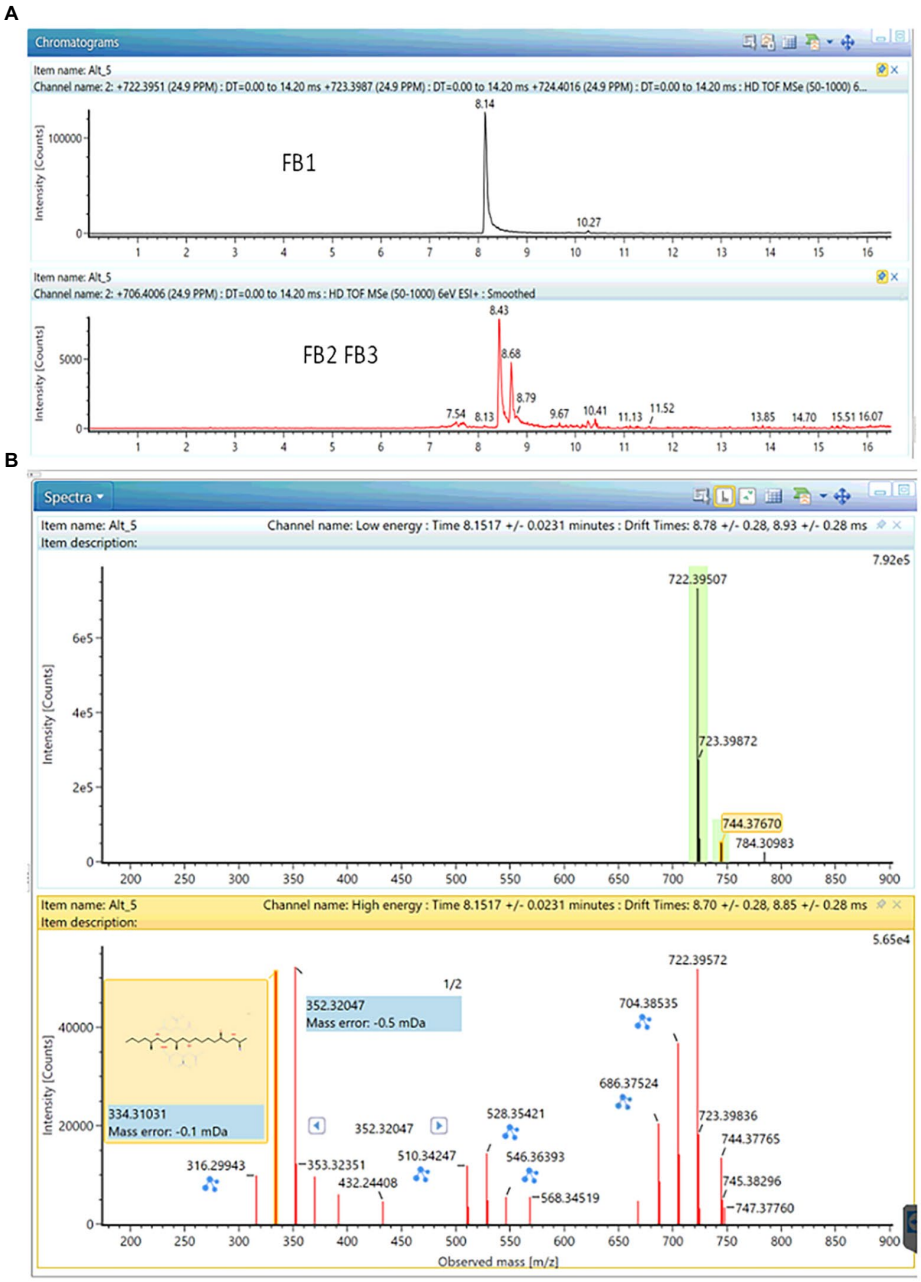
Production of FB_1_ together with small traces of FB_2_ and FB_3_ in ITEM18910 strain. **(A)** Chromatogram; **(B)** MS and fragmentation spectra (mass error: −0.9 ppm; Δr_T_: 0.01 min; ΔCCS: −0.30).

Based on the aforementioned approach, the production of small amounts of fumonisin B1 by four out of five *Curvularia* strains (ITEM18909, ITEM18910, ITEM18912, ITEM18913) was detected (see Figure 3). The annotation was further confirmed through comparison with the analytical standard, returning a mass error of-0.9 ppm; a shift in retention time of 0.12% as well as a ΔCCS% of-0.30. All these parameters fall well within the quality criteria for annotation.

Small amounts of FB2 and FB3 have been found in cultures of ITEM18910 as well (see Figure 3; mass error: −1.3 ppm for both FB2 and FB3; Δr_T_%: 2.7 and 0.1% for FB2 and FB3 respectively; ΔCCS%: −1.48% and-0.84% for FB2 and FB3 respectively).

A preliminary comparison of the production capability was performed based on the amount detected in the five strains after the same extraction, ITEM18910 being the highest producer. The absence of FB1 in ITEM18911 could be due to a lower production, falling below the instrumental sensitivity. Further studies should be carried out to further confirm and better understand the capability to synthesize FB1 in *C spicifera* strains.

### Pathogenicity test

**Figure 4:**
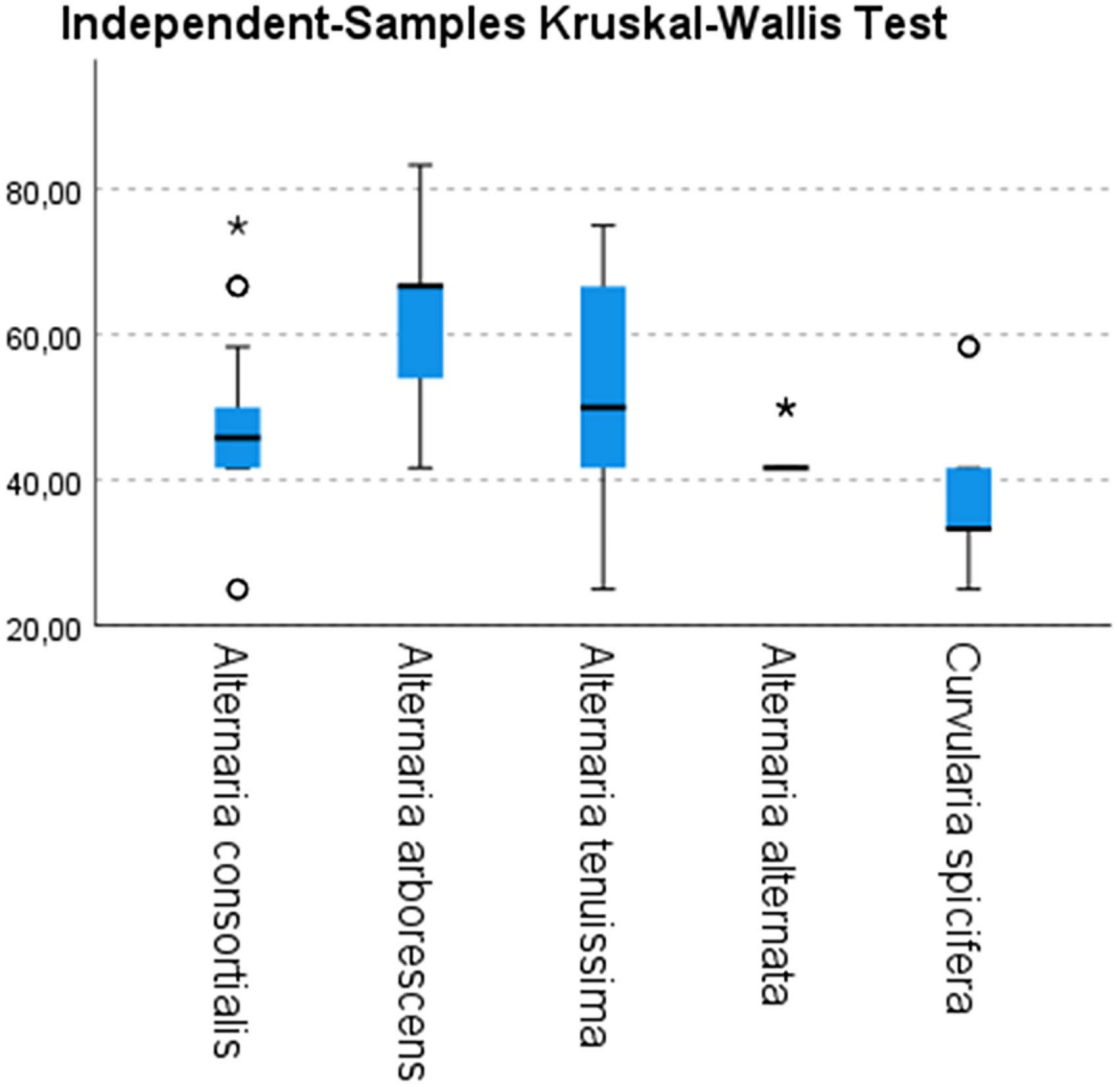
Disease severity index (vertical axes) calculated on inoculated date palm plantlets with strains belonging to different species of *Alternaria* (10 *A. consortialis* strains, 9 *A. arborescens* strains, 17 *A. tenuissima* strains, 3 *A. alternata* strains) and to *Curvularia spicifera* (3 strains). Differences among species have been statistically evaluated through independent-samples Kruskal–Wallis test: *χ*^2^ (*df* = 4, *N* = 126) = 28.575; *p* < 0.05. Box plots are displayed, where the bold line indicates the median per group, the box represents 50% of the values, and horizontal lines show minimum and maximum values of the calculated nonoutlier values; asterixes and open circles indicate outlier values.

All inoculated plantlets showed the typical symptoms of leaf spot disease whereas the control plantlets did not show any symptoms up to 3 months after inoculation. However, symptomatic plantlets showed different degrees of disease severity (Figure 4).

The typical leaf spot symptoms developed 10 days after inoculation. In particular, necrotic spots appeared on the inoculated plants 5–7 days after inoculation, and spread along the entire leaves and stems within 15 days. Among the fungal species tested, *A. arborescens* showed a higher DSI than other species, ranging from 52.7 to 72.2 (mean value 62) followed by *A. tenuissima* (mean value 52) and *A. consortialis* (mean value 47.8), as showed in Figure 4. The *A. alternata* and *C. spicifera* stains showed a lower degree of severity with DSI ranging between 41.6 and 48, or 33.3 and 47.2, respectively (Figure 4).

## Discussion

### Species identification

In this study, we report for the first time an extensive study on the *Alternaria* species occurring on date palm leaves showing leaf spot symptoms, in Tunisia. In recent years, a high incidence of leaf spot disease was recorded on date palm (Namsi et al., 2021). In the period 2017–2019, we inspected palm date plantations located in Djerib, the most intense area of date production in Tunisia. Leaf spot disease was detected in all localities investigated, with incidence values up to 22% in Mides area (data not shown). Although traditional varieties such as *Beser Hlou*, *Kentikhi*, and *Alig* are cultivated in this region, *Deglet Nour* is the most popular and widely cultivated variety in Tunisia. However, this variety is highly susceptibile to fungal pathogens including *Fusarium oxysporum* f. sp. *albedinis* and a range of fungal species associated with foliar disease (Matheron and Benbadis, 1990; Hamza et al., 2015). The most important pathogens associated to leaf spot disease are *Alternaria* species. Based on multi-locus sequence analyses, all *Alternaria* strains considered in this study were assigned to two main Sections: *Ulocladioides* that included only *A. consortialis* species, and *Alternaria* section, that included 3 well-separated sub-clades corresponding to the formerly described *A. alternata*, *A. arborescens* and *A. tenuissima* morpho-species. Several studies report *Alternaria* species among the most important phytopathogenic fungi associated with date palm leaf spot disease. However, in almost all studies *Alternaria* strains were identified only based on morphological traits. In Oman, sequence analyses of *ITS* region showed that *A. arborescens* and *A. alternata* were the most common *Alternaria* species associated with leaf spots of date palms (Al-Sadi et al., 2012). However, *ITS* region alone is not very informative to identify *Alternaria* species (Woudenberg et al., 2013). Indeed, in the same country, two subsequent extensive studies, based on the sequence analyses of the *ITS*, *gpd*, *tef* and RNA polymerase II subunit informative genes, confirmed *A. alternata* and *A. arborescens* as the most frequent species isolated from symptomatic date palm leaves, but also revealed the presence of two other species included in the *Alternaria* section: *A. burnsii* and *A. tomato* species (Al-Nadabi et al., 2018, 2020). In addition to the above mentioned species, other species have been associated with leaf blight in Tunisia. Ben Chobba et al. (2013) identified also *A. gaisen* (included in *Alternaria* Section) by using *ITS1* and *ITS4* genes, whereas Namsi et al. (2021) identified *A. mali*, by using *ITS*, *gpd*, *CaM*, and *Alt-a1* genes. However, in other studies, *A. mali* was proposed as synonymous to *A. alternata* species (Woudenberg et al., 2015), whereas, according to Somma et al. (2019), *A. mali* was phylogenetically more similar to *A. gaisen*, both species being distinguishable from *A. alternata*. These incongruences could be likely due to the different genomic regions considered for phylogenetic analysis, which suggests that it would be beneficial to establish a common pool of genes and procedures to be unanimously adopted by the scientific community dealing with *Alternaria* genus taxonomy, to better define the phylogenetic relationships among species. On the other hand, the previously described morpho-species *A. alternata* and *A. tenuissima* were compared according to their whole genomes and transcriptomes (Woudenberg et al., 2015), and for multi-gene sequences (Somma et al., 2019). From such comparisons, the two species were both included in *Alternaria* section by Woudenberg et al. (2015) and Somma et al. (2019) who merged them in the same species, namely *A. alternata*. Furthermore, in both papers, *A. arborescens* formed a distinct clade from *A. alternata*, confirming the data here reported. In our study, we report for the first time the occurrence of *A. consortialis* (Section *Ulocladoidies*) as a cause of disease symptoms on date palm leaves. Also, we report here for the first time, the pathogenicity of *C. spicifera* on date palm leaves in Tunisia. This species has been never reported on date palm, although many other species of the genus *Curvularia* have been reported to occur on date palm symptomatic leaves, such as *C. subpapendrofii* in Iraq and Oman (Abass et al., 2007; Al-Nadabi et al., 2020), *C. verruculosa* and *C. hawaiiensis* in Oman (Al-Nadabi et al., 2020), and *C. palmivora* in Egypt (Arafat et al., 2021).

### Pathogenicity test

The 3 strains of *C. spicifera* tested for pathogenicity showed to be all moderately aggressive in our bioassay, carried out on the *Deglet Nour* variety. They were the least aggressive in our tests compared the species of *Alternaria* tested. These data are in contrast with Arafat et al. (2021) that showed *C. spicifera* and *C. palmivora* were highly aggressive on date palm in Egypt. In addition, another species of the genus *Curvularia*, *C. verruculosa*, proved to be highly aggressive on date palm leaves (Al-Nadabi et al., 2020). Among the *Alternaria* strains tested, those belonging to *A. consortialis* showed to be pathogenic at a moderate level compared the other *Alternaria* species tested, where the most pathogenic strains were those belonging to *A. arborescens*, followed by *A. tenuissima*, while the less pathogenic were the strains belonging to *A. alternata*. These data agree with Al-Nadabi et al. (2020) who reported that *A. alternata*, isolated in Oman from date palm, was weakly pathogenic on date palm leaves assay. On the other hand, they reported that strains of *A. burnsii* and *A. tomato* were highly pathogenic, but we did not detect these species in our study (Al-Nadabi et al., 2020).

### Mycotoxin profile

In addition to their pathogenicity, which can lead to a reduced productivity, all *Alternaria* species identified in this survey are also of concern since they can produce a wide range of mycotoxins (Logrieco et al., 2009). Their occurrence on date palm plants indicates that a contamination of fruit at the harvest in the field and its by-products in postharvest can occur. Since we showed a high occurrence of toxigenic *Alternaria* species in plants, monitoring environmental conditions in the field suitable for mycotoxin production in planta by the *Alternaria* species is important in order to manage and avoid an eventual, although unlikely, contamination of fruits. Many date-based products such as pickles, chutney, jam, jelly, date-in-syrup, date butter, candy, date bars and confectionary products are consumed in different parts of the world, including Tunisia. Some evidence of contamination by *Alternaria* in date palm fruits are available (Palou et al., 2016; Piombo et al., 2020). Therefore, as a consequence, this contamination could cause risk for consumers, but could also interfere with date fruits industrial processing. Our chemical and phylogenetic results showed that most of the identified *Alternaria* strains belong to toxigenic species and could produce all mycotoxin tested, ALT, AOH, AME, TEN, and TA, with the exception of *A. consortialis* strains, which did not produce any mycotoxin tested. Studies have shown that TA was responsible for some changes in the esophageal mucosa of mice (Yekeler et al., 2001) and was reported as cytotoxic, phytotoxic, antitumoral, antiviral, antibiotic and antibacterial compound (Asam et al., 2013). Solhaug et al. (2015) reported AOH and AME as the most detected mycotoxins in food and feed products, both identified as genotoxic and mutagenic with immune modulating effects. Recently, Huybrechts et al. (2018) associated *Alternaria* mycotoxins also with colon rectal cancer in humans. Due to this wide range of toxic effects of *Alternaria* mycotoxins, the need for correct identification of *Alternaria* species is a key aspect, since many species have a specific mycotoxin profile. Therefore, accurate risk assessment is strongly linked to the use of reliable and advanced diagnostic tools. The extended production of the *Alternaria* mycotoxins by several *Alternaria* strains tested in this work, that were able to produce all mycotoxins analyzed, shows that a co-occurrence of *Alternaria* mycotoxins on date palm plants would be likely, indicating an increased risk for the contamination of final products. In addition, some of the *Alternaria* mycotoxins can play a crucial role in pathogenicity processes, acting as virulence and colonization factors. For instance, Wenderoth et al. (2019) demonstrated that the AOH biosynthesis pathway was responsible for the production of at least five different secondary metabolites and that AOH facilitates growth of *A. alternata* on various fruits and leaves during infection. In addition, TA is the essential requirement for successful colonization and disease development in host leaves of *Ageratina adenophora* (Shi et al., 2021). The fact that many strains analyzed in this work can produce both AOH and TA triggers further investigations on the role that these secondary metabolites can play in *Alternaria* aggressiveness against date palm plants. Finally, the strains of *C. spicifera* proved to be able to produce FBs. How widely distributed this pathogen is on date palm and how extended is the ability of *C. spicifera* to produce FBs need to be further investigated by testing more strains. Specific investigations are in progress, in order to evaluate the genetic structure of the biosynthetic gene pathway responsible of FBs production in this species. *Curvularia spicifera* species have been detected also on strawberry in Iran (Ayoubi et al., 2017), on citrus fruits in south Italy (Garganese et al., 2015), on date palm fruit in Saudi Arabia (Al-Sheikh, 2009). Although in these studies, the authors did not evaluate the capability of these strains to produce mycotoxins, our results should trigger further studies aimed to evaluate the toxigenic risk occurring on the final products of these important crops. Controversial reports exist on the ability of the FBs to be phytotoxic toward plants. In particular, the AAL toxin, structurally identical to FBs, was proved to be a specific toxin required to cause pathogenicity in tomato colonized by *A. alternata forma specialis lycopersici* (sinonym. *A. arborescens*). On the other hand, Arias et al. (2012) proved that FBs play a role in virulence but also that FB production is not necessary or sufficient for virulence on maize seedlings. Therefore, further experiments are required to test FB phytotoxicity on date palm plants and better understand their role in the pathogenesis process, related to *C. spicifera*.

In conclusion, our studies have provided new insights on the identification of new pathogenic *Alternaria* and *Curvularia* species affecting date palm, in Tunisia. The phylogeny of the *Alternaria* species here performed has confirmed that *A. arborescens* formed a distinct clade from *A. alternata*, and that the previously described morpho-species *A. alternata* and *A. tenuissima* should be merged in the same species, namely *A. alternata*. A high mycotoxin concern in date palm is due to wide ability of many of the *Alternaria* strains tested to produce all mycotoxins analyzed, and in-depth investigation on the role of *Alternaria* toxins in colonization of date palm is required. Finally, the ability of the *Curvularia* strains to produce the highly harmful FBs triggers further studies to better investigate their possible role in date palm colonization and eventual risk for consumers.

## Data availability statement

The data presented in the study are deposited in the GenBank repository, accession numbers ON688310 - ON688454.

## Author contributions

AR: investigation, formal analysis, data curation, writing-original draft. MM: methodology, formal analysis, investigation, data curation, writing-original draft, writing-review and editing. SS: methodology, formal analysis, investigation, data curation, writing-original draft. FC: investigation, formal analysis. CD: conceptualization and methodology. LR: methodology, validation and writing-original draft. AS: methodology and data curation. AL: funding acquisition. AN: investigation, methodology. RG: investigation. SW: supervisor. AM: project administration, writing-original draft, writing-review and editing, funding acquisition. All authors contributed to the article and approved the submitted version.

## Conflict of interest

The authors declare that the research was conducted in the absence of any commercial or financial relationships that could be construed as a potential conflict of interest.

## Publisher’s note

All claims expressed in this article are solely those of the authors and do not necessarily represent those of their affiliated organizations, or those of the publisher, the editors and the reviewers. Any product that may be evaluated in this article, or claim that may be made by its manufacturer, is not guaranteed or endorsed by the publisher.

## Supplementary material

Supplementary Figure S1 Extracted ions chromatogram (XIC) showing the absence of FB1 in blank rice medium.
