# Supplementary figures and images for: Phylogeny and mycotoxin profiles of pathogenic Alternaria and Curvularia species isolated from date palm in southern Tunisia
Source: Front Microbiol. 2022 Nov 7;13:1034658. doi: 10.3389/fmicb.2022.1034658 (PMC9677452; doi:10.3389/fmicb.2022.1034658)

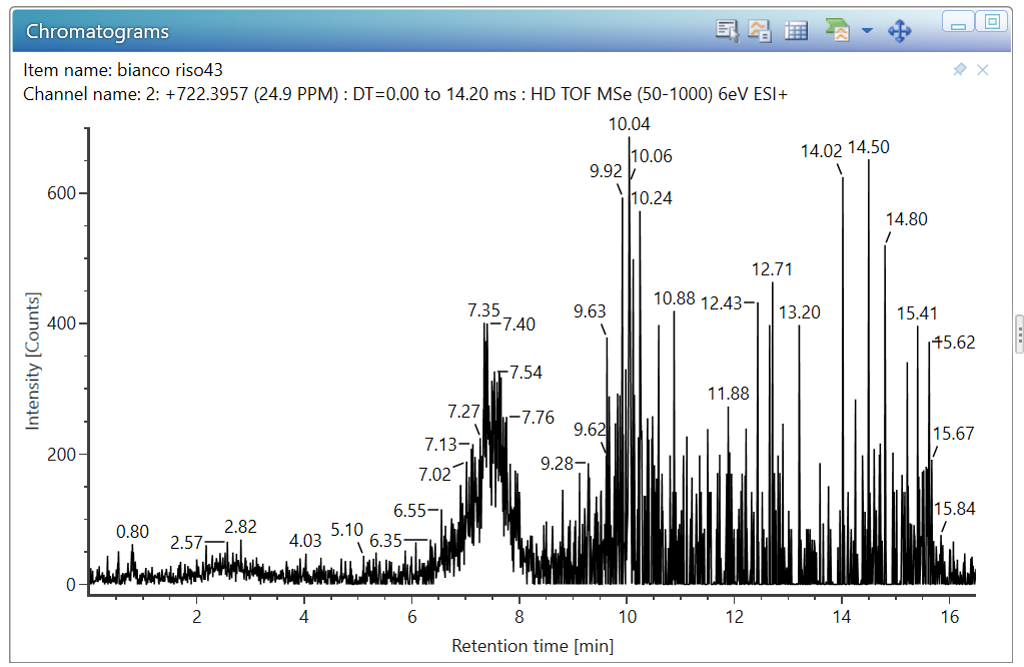

Supplement: Supplementary Figure S1 — Extracted ions chromatogram (XIC) showing the absence of FB1 in blank rice medium. [file Image_1.TIF]
