# Supplementary material for: Phylogeny and mycotoxin profiles of pathogenic Alternaria and Curvularia species isolated from date palm in southern Tunisia
Source: Front Microbiol. 2022 Nov 7;13:1034658. doi: 10.3389/fmicb.2022.1034658 (PMC9677452; doi:10.3389/fmicb.2022.1034658)
Supplement: Supplementary file 2 [file Table_1.docx]

**Table 1S.** Pathogenicity of 39 *Alternaria* strains and 3 *Curvularia* *spicifera* strains selected for pathogenicity assay. For each experiment, 3 replications consisting of 3 plantlets per replicate were considered.

| Strain | *Alternaria* species | Disease Severity Index |
| --- | --- | --- |
| A3 | *A.consortialis* | 41.7^ab^ |
| A6 | *A.consortialis* | 41.7^ab^ |
| A36 | *A.consortialis* | 50^ab^ |
| A38 | *A.consortialis* | 55.5^ab^ |
| Alt1559 | *A.consortialis* | 41.7^ab^ |
| Alt1553 | *A.consortialis* | 44^ab^ |
| Alt1565 | *A.consortialis* | 58.2^ab^ |
| Alt1568 | *A.consortialis* | 42^ab^ |
| A33 | *A.consortialis* | 42^ab^ |
| Alt1571 | *A.consortialis* | 61.1^ab^ |
|  | **Mean value** | **47.8** |
| A8 | *A.arborescens* | 66.6^ab^ |
| A14 | *A.arborescens* | 66.6^ab^ |
| A16 | *A.arborescens* | 60.6^ab^ |
| Alt1558 | *A.arborescens* | 52.7^ab^ |
| Alt1575 | *A.arborescens* | 72.2^a^ |
| Alt1569 | *A.arborescens* | 61.6^ab^ |
| Alt1550 | *A.arborescens* | 63.8^ab^ |
| Alt1570 | *A.arborescens* | 55.5^ab^ |
| A37 | *A.arborescens* | 58.2^ab^ |
|  | **Mean value** | **62** |
| A23 | *A.tenuissima* | 58.2^ab^ |
| A26 | *A.tenuissima* | 55.5^ab^ |
| A30 | *A.tenuissima* | 41.6^ab^ |
| A4ALT | *A.tenuissima* | 50^ab^ |
| Alt1554 | *A.tenuissima* | 55.5^ab^ |
| Alt1557 | *A.tenuissima* | 47.2^ab^ |
| Alt1576 | *A.tenuissima* | 61.1^ab^ |
| Alt1579 | *A.tenuissima* | 47.2^ab^ |
| Alt1551 | *A.tenuissima* | 41.6^ab^ |
| A13 | *A.tenuissima* | 52.7^ab^ |
| Alt1560 | *A.tenuissima* | 60.6^ab^ |
| Alt1577 | *A.tenuissima* | 61.1^ab^ |
| Alt1567 | *A.tenuissima* | 66.6^ab^ |
| Alt1580 | *A.tenuissima* | 60.6^ab^ |
| Alt1556 | *A.tenuissima* | 41.7^ab^ |
| Alt1564 | *A.tenuissima* | 41.7^ab^ |
| Alt1574 | *A. tenuissima* | 41.7^ab^ |
|  | **Mean value** | **52** |
| A12 | *A. alternata* | 48^ab^ |
| A19 | *A. alternata* | 41.6^ab^ |
| Alt1563 | *A. alternata* | 41.6^ab^ |
|  | **Mean value** | **43.7** |
| ITEM18909 | *C. spicifera* | 33.3^b^ |
| ITEM18912 | *C. spicifera* | 47.2^ab^ |
| ITEM18913 | *C. spicifera* | 38.8^ab^ |
|  | **Mean value** | **39.7** |
